# Phenotypic and Genomic Analysis of Swine-Derived *Bacillus subtilis* 1BP1Co1 as a Functional Probiotic Candidate to Mitigate *Escherichia coli*-Associated Diarrhea

**DOI:** 10.3390/ani16162449

**Published:** 2026-08-07

**Authors:** Rumpa Jutakanoke, Warunya Chakritbudsabong, Wongsakorn Phongsopitanun, Wuttichai Mhuantong, Jirasin Koonthongkaew, Noppadon Siangpro, Songkran Chuakrut, Sasitorn Rungarunlert

**Affiliations:** 1 Department of Microbiology and Parasitology, Faculty of Medical Science, Naresuan University, Phitsanulok 65000, Thailand; rumpaj@nu.ac.th (R.J.); songkranc@nu.ac.th (S.C.); 2 Department of Pre-Clinic and Applied Animal Science, Faculty of Veterinary Science, Mahidol University, Nakhon Pathom 73170, Thailand; warunya.cha@mahidol.ac.th; 3 Laboratory of Cellular Biomedicine and Veterinary Medicine, Faculty of Veterinary Science, Mahidol University, Nakhon Pathom 73170, Thailand; 4 Department of Biochemistry and Microbiology, Faculty of Pharmaceutical Sciences, Chulalongkorn University, Bangkok 10330, Thailand; wongsakorn.p@chula.ac.th; 5 National Center for Genetic Engineering and Biotechnology (BIOTEC), National Science and Technology Development Agency (NSTDA), Pathum Thani 12120, Thailand; wuttichai.mhu@biotec.or.th; 6 Department of Microbiology, Faculty of Sciences, Chulalongkorn University, Bangkok 10330, Thailand; jirasin.k@chula.ac.th; 7 Research Unit in Bioconversion/Bioseperation for Value-Added Chemical Production, Chulalongkorn University, Bangkok 10330, Thailand; 8 Office of Disease Prevention and Control, Region 3 Nakhon Sawan, Nakhon Sawan 60000, Thailand; noppadonsiangpro@gmail.com

**Keywords:** swine production, post-weaning diarrhea, *Escherichia coli*, probiotics, *Bacillus subtilis*

## Abstract

Post-weaning diarrhea caused by pathogenic *Escherichia coli* is a significant economic challenge in global swine production. Rising antimicrobial resistance linked to traditional antibiotics necessitates safe, sustainable alternatives. This study isolated and characterized host-associated *Bacillus* species from the gastrointestinal tracts of healthy pigs to evaluate their probiotic potential. Fifty-nine *Bacillus* isolates were screened for safety, gastrointestinal tract survivability, mucosal adhesion, and antimicrobial efficacy. Several isolates demonstrated robust tolerance to simulated gastrointestinal stress (acidic pH and bile salts), non-hemolytic profiles, and strong mucosal adhesion. Multiple isolates displayed inhibitory activity against pathogenic *Escherichia coli* in vitro. The most promising candidate, *Bacillus subtilis* 1BP1Co1, underwent Nanopore whole-genome sequencing. Genomic profiling confirmed its safety, revealing a complete absence of known virulence factors and high-risk antimicrobial resistance genes. Furthermore, genome annotation identified multiple biosynthetic gene clusters responsible for the production of antimicrobial secondary metabolites, including surfactin, fengycin, bacillaene, and subtilosin A, which mechanistically supports its pathogen-inhibitory properties. Collectively, these findings highlight indigenous *Bacillus* strains, particularly *Bacillus subtilis* 1BP1Co1, as promising probiotic candidates with the potential to be developed to mitigate *Escherichia coli*-associated enteric disorders in swine production systems.

## Simple Summary

Post-weaning diarrhea in pigs, often caused by pathogenic *Escherichia coli*, is a major problem in the global swine industry. Given the increasing global pressure to reduce the use of antibiotic growth promoters, there is an urgent need for effective and safe alternatives to prevent these infections. In this study, we isolated *Bacillus* bacteria from the digestive tracts of healthy pigs and evaluated their potential as probiotics. Through this process, we identified a highly promising strain, *Bacillus subtilis* 1BP1Co1. Laboratory tests and whole-genome sequencing confirmed that this strain shows a favorable in vitro safety profile, survives the harsh conditions of the digestive system, adheres well to the gut lining, and effectively inhibits the growth of pathogenic *Escherichia coli*. These findings suggest that *Bacillus subtilis* 1BP1Co1 is a promising probiotic candidate with the potential to help prevent diarrhea in the swine industry and reduce reliance on antibiotics in pig farming.

## 1. Introduction

Post-weaning diarrhea (PWD) remains one of the most economically significant enteric diseases in pig production systems worldwide. Among the primary etiological agents, *Escherichia coli* (*E. coli*) is recognized as a major pathogen responsible for intestinal inflammation, epithelial barrier disruption, impaired nutrient absorption, and growth retardation in weaned pigs. The traditional reliance on antibiotic growth promoters (AGPs) to control *E. coli*-associated diarrhea has contributed to the global rise in antimicrobial resistance (AMR), prompting urgent efforts to identify sustainable and safe alternatives.

Probiotic supplementation has emerged as a promising strategy to mitigate enteric infections through competitive exclusion, enhancement of gut barrier integrity, modulation of immune responses, and secretion of antimicrobial compounds. In particular, the spore-forming genus *Bacillus* has gained increasing attention due to its remarkable environmental resilience and functional versatility. The ability of *Bacillus* to form endospores enables survival under harsh feed-processing conditions and gastrointestinal stress, ensuring the delivery of viable cells to the intestine [1].

Several studies demonstrate the beneficial effects of *Bacillus* supplementation in weaned pigs challenged by enteric pathogens. Guo et al. (2006) reported that *Bacillus subtilis* MA139 improved growth performance and feed efficiency in weaned pigs, confirming its probiotic potential through both in vitro screening and in vivo validation [2]. Similarly, dietary inclusion of *B. subtilis* strains significantly reduced diarrhea incidence and improved growth performance during the post-weaning period [3,4]. Improvements in villus height, tight junction integrity, and immune modulation have been associated with enhanced resistance to intestinal pathogens, including pathogenic *E. coli* [4,5]. These findings suggest that *B. subtilis* contributes not only to growth promotion but also to intestinal health stabilization during pathogen exposure.

In addition to immunomodulatory effects, *Bacillus* exhibits direct antimicrobial activity. Certain strains produce bacteriocins and secondary metabolites capable of inhibiting Gram-negative pathogens, including *E. coli* [6,7]. Genomic and functional characterization studies have further demonstrated the antimicrobial and probiotic properties of host-derived strains, such as *B. subtilis* AJQ03 isolated from the intestinal tract of *Anguilla japonica*, which exhibited pathogen inhibition, absence of hemolytic activity, and strong colonization potential [8]. Genomic analysis of pig-derived *Bacillus* has revealed biosynthetic gene clusters responsible for antimicrobial peptide production, supporting their functional role in pathogen suppression [7]. Such antimicrobial capacity is particularly relevant in the context of enterotoxigenic *E. coli* (ETEC) colonization, where competitive inhibition and antimicrobial secretion may reduce pathogen load in the gut. These findings support the importance of host-associated strain selection in probiotic development.

Furthermore, advances in strain development extend beyond laboratory screening. Cao et al. (2025) described a comprehensive workflow from indigenous strain screening to pilot-scale fermentation of *B. subtilis* YZ01, emphasizing safety assessment, enzymatic profiling, and production optimization for large-scale application [9]. Such integrated approaches strengthen the translational potential of probiotic candidates for industrial use. Comparative studies have also demonstrated that *B. subtilis* supplementation can serve as an effective alternative to AGPs such as carbadox in improving performance parameters and intestinal health in weaned pigs [10]. This is especially important given the regulatory phase-out of antibiotic growth promoters in many countries. The probiotic-mediated improvement in digestive enzyme activity and nutrient digestibility may further contribute to reduced substrate availability for opportunistic pathogens such as *E. coli* [11].

Despite the growing evidence supporting *Bacillus* as a probiotic candidate, probiotic efficacy remains highly strain-specific. Therefore, comprehensive safety assessment—including hemolytic activity evaluation—alongside characterization of enzyme production and antimicrobial activity is essential prior to practical application [1,6]. Strains derived from host-associated environments may exhibit enhanced ecological fitness and colonization capacity, potentially increasing their effectiveness against enteric pathogens [1].

The present study aimed to isolate and characterize host-associated *Bacillus* strains and evaluate their probiotic potential with particular emphasis on their capacity to inhibit enteric pathogens such as *E. coli*. Specifically, we assessed safety-related properties, including hemolytic activity, extracellular enzyme production, antimicrobial activity against selected pathogens, and phenotypic characteristics associated with gastrointestinal survivability. By integrating safety evaluation with functional screening, this study seeks to determine the suitability of *Bacillus* strains as a sustainable probiotic alternative for mitigating *E. coli*-associated enteric disorders in swine production systems.

## 2. Materials and Methods

### 2.1. Ethical Approval

The study protocol was reviewed by the Institutional Animal Care and Use Committee (IACUC) of the Faculty of Veterinary Science, Mahidol University. Because the study exclusively utilized postmortem intestinal and fecal samples obtained from porcine carcasses already processed for commercial food production at a registered slaughterhouse, the IACUC determined that formal animal ethics approval was not required. No live animals were used, and no experimental procedures were performed on animals prior to slaughter; therefore, the study complies with the ethical guidelines for the use of animal-derived by-products in research.

### 2.2. Sample Collection

**Figure 1:**
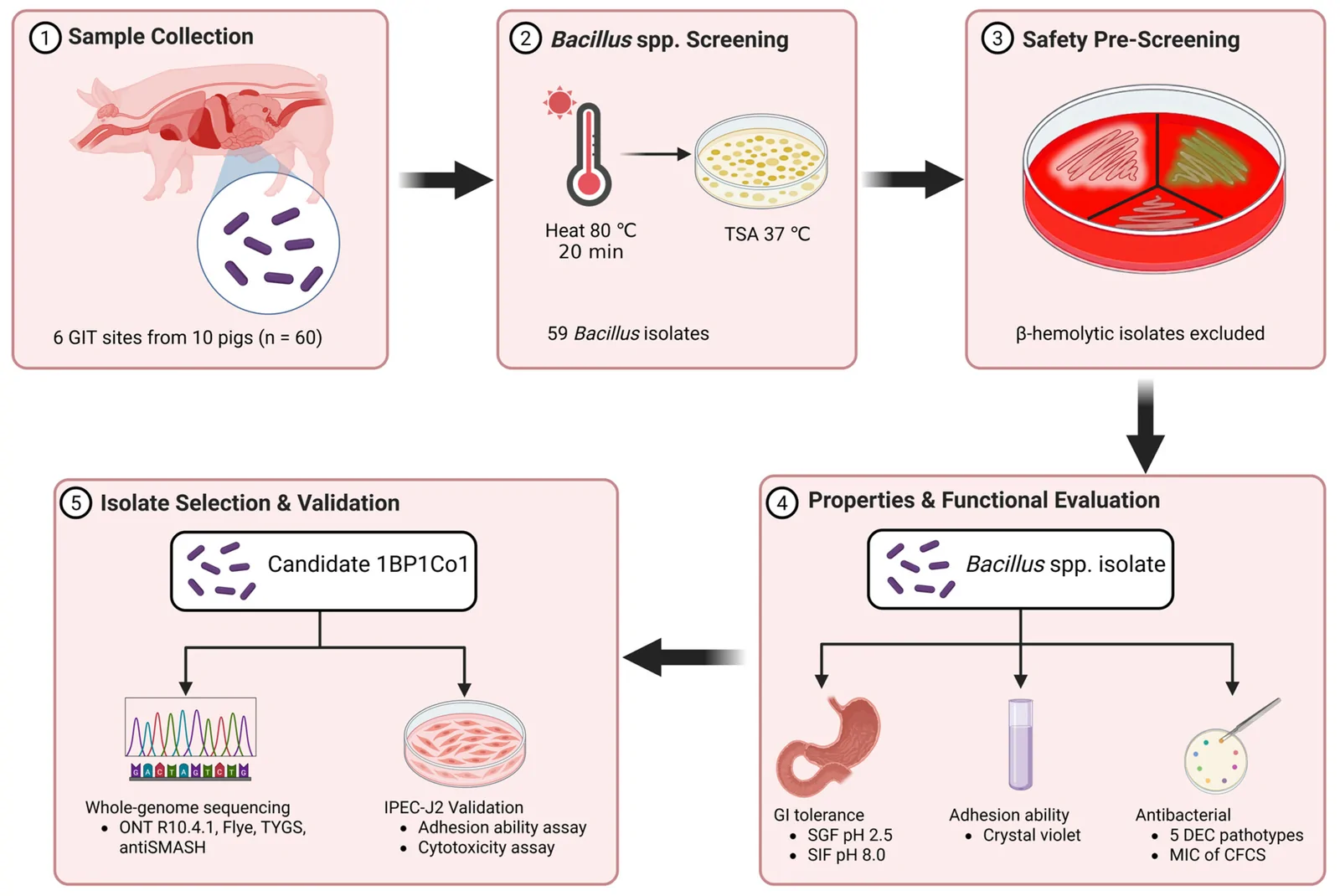
Experimental workflow for the isolation, screening and characterization of swine-derived *Bacillus* spp. Samples from six gastrointestinal sites of ten pigs (*n* = 60) were heat-selected for spore-formers, yielding 59 *Bacillus* isolates. After exclusion of β-hemolytic isolates, three parallel screens (gastrointestinal tolerance, surface attachment, and antibacterial activity against five diarrhoeagenic *E. coli* pathotypes) informed selection of candidate 1BP1Co1, which was validated on IPEC-J2 cells and by whole-genome sequencing.

A total of 60 biological samples were obtained from 10 crossbred pigs (*Sus scrofa*; Landrace × Large White × Duroc; 5 barrows and 5 gilts; aged approximately 24 weeks) at two commercial slaughterhouses in Nakhon Pathom and Pathum Thani, Thailand. Both facilities operate under certified Good Manufacturing Practices (GMP). To ensure a comprehensive representation of the gastrointestinal microbiota, samples were harvested from six distinct sites per animal: five intestinal segments (duodenum, jejunum, ileum, cecum, and mid-colon) and distal feces. The 60 samples corresponded to six anatomical sites sampled from each of 10 animals (*n* = 10 per site). All samples were collected under aseptic conditions to prevent environmental contamination and cross-contamination among samples. For intestinal samples, appropriately sized tissue segments were excised and divided into three portions to serve as replicates. Each portion was placed into a sterile 50 mL conical tube containing Tryptone Soya Broth (TSB) supplemented with 30% (*w*/*v*) glycerol to preserve microbial viability during storage and transportation (Figure 1).

Fecal samples were collected separately in sterile containers and processed under the same aseptic conditions. All samples were transported to the laboratory under temperature-controlled conditions (on ice) and subsequently stored at −20 °C until further processing.

### 2.3. Isolation of Bacillus spp.

Isolation of *Bacillus* spp. was performed using a heat-selection method to enrich spore-forming bacteria. Briefly, 0.5 g of each sample was mixed with 1 mL of sterile 0.85% (*w*/*v*) NaCl solution in a 1.5 mL microcentrifuge tube. The sample suspension was subjected to heat treatment at 80 °C for 20 min to eliminate non-spore-forming vegetative bacteria. Immediately after heating, the tubes were placed in ice water for 1–2 min to rapidly terminate the heat treatment.

The treated suspension was spread onto Tryptic Soy Agar (TSA) plates and incubated at 37 °C for 18–24 h. Colonies with distinct morphological characteristics, including differences in color, size, shape, margin, and surface texture, were selected and purified by repeated streaking on TSA plates until pure cultures were obtained.

### 2.4. Microscopic Observation and Spore Staining

Pure cultures were examined microscopically to observe cellular morphology. Smears were prepared from single colonies and subjected to Gram staining. Observations were made under a light microscope at 1000× magnification.

Spore formation was confirmed using the Schaeffer–Fulton spore staining method. Briefly, heat-fixed smears were flooded with malachite green and heated over steam for approximately 5 min. The slides were then rinsed with distilled water and counterstained with safranin for 30–60 s. Under this staining protocol, endospores stain green, whereas vegetative cells stain red or pink.

### 2.5. Colony Morphology Characterization

Colony morphology was recorded after incubation on TSA plates at 37 °C for 18–24 h. Characteristics including colony color, shape, margin, surface texture, and elevation were documented. Cellular arrangements observed under the microscope, such as chains or pairs, were also recorded for each isolate.

### 2.6. Hemolytic Activity Assay

Hemolytic activity was evaluated using blood agar plates supplemented with 5% (*v*/*v*) sheep blood. *Bacillus* isolates were streaked onto the plates and incubated at 37 °C for 24–48 h. Hemolytic patterns were classified as β-hemolysis (a clear zone of complete red blood cell lysis around the colonies), α-hemolysis (a greenish zone of partial lysis), or γ-hemolysis (no visible hemolysis). Isolates exhibiting β-hemolysis were excluded from further probiotic property evaluation due to potential safety concerns.

### 2.7. Antimicrobial Activity Against Pathogenic Bacteria

The antibacterial activity of *Bacillus* isolates against pathogenic *E. coli* groups was evaluated using the agar slab assay. The pathogenic *E. coli* pathotypes used in this study included enteroaggregative *E. coli* (EAEC), enterohemorrhagic *E. coli* (EHEC), enteroinvasive *E. coli* (EIEC), enterotoxigenic *E. coli* (ETEC), and enteropathogenic *E. coli* (EPEC). Each pathogenic *E. coli* culture was adjusted to approximately a 0.5 McFarland standard and uniformly spread onto Mueller–Hinton agar (MHA) plates.

Agar slabs containing actively grown *Bacillus* isolates were aseptically transferred onto the surface of MHA plates previously inoculated with each pathogenic *E. coli* pathotype. The plates were incubated at 37 °C for 18–24 h. After incubation, antibacterial activity was assessed by measuring the diameter of the inhibition zone around each agar slab in millimeters. A positive control and a negative control were included in each experiment to ensure the validity of the assay.

The agar slab assay was used as the primary screening method to identify *Bacillus* isolates with antibacterial activity and to select representative isolates for subsequent Minimum inhibitory concentration (MIC) determination. However, isolate selection was not based solely on the largest inhibition-zone diameter. Instead, candidates were selected based on their overall antibacterial profile, including their broad inhibitory spectrum across multiple pathogenic *E. coli* pathotypes, consistency of inhibition, and suitability for quantitative confirmation by MIC testing.

#### Minimum Inhibitory Concentration (MIC) of Cell-Free Culture Supernatants

MIC testing was performed to quantitatively confirm the antibacterial activity of selected *Bacillus* isolates. Cell-free culture supernatants (CFCSs) were prepared by culturing each isolate in TSB at 37 °C for 24–48 h, centrifuging at 10,000× *g* for 15 min, and filtering the supernatant through a sterile 0.22 µm membrane. MIC testing was conducted using a modified broth microdilution method based on the principle of CLSI M07 and ISO 20776-1 [12,13]. Two-fold serial dilutions of each CFCS were prepared to obtain nominal CFCS dilution levels of 100, 50, 25, 12.5 and 6.25% (*v*/*v*). Each pathogenic *E. coli* culture was adjusted to a 0.5 McFarland standard and diluted in Mueller–Hinton broth (MHB) before inoculation into the wells containing CFCS dilutions.

Growth control wells containing pathogenic *E. coli* without CFCS and sterility control wells containing sterile medium without a bacterial inoculum were included in each assay. The plates were incubated at 37 °C for 24 h. The MIC was defined as the lowest nominal CFCS dilution level showing no visible bacterial growth after incubation. Optical density at 600 nm (OD_600_) values were recorded as supporting data for MIC interpretation, alongside visual growth assessment of the test and control wells. Because CFCS is a complex culture-derived preparation and not a purified antimicrobial compound with a defined mass concentration, MIC values were expressed as nominal CFCS dilution levels (% *v*/*v*) rather than as final concentrations of purified active compounds.

### 2.8. Gastrointestinal Stress Tolerance 

#### 2.8.1. Tolerance to Simulated Gastric Conditions

Each isolate was purified by repeated streaking on agar plates until a single colony was obtained. A single colony was then inoculated into TSB and incubated at 37 °C for 18–24 h. Bacterial cells were harvested by centrifugation and washed twice with sterile 0.85% (*w*/*v*) sodium chloride (NaCl) solution to remove residual growth medium and metabolites. The cell suspension was subsequently adjusted to approximately a 0.5 McFarland standard (≈1.5 × 10^8^ CFU/mL) before use in the assays. For the simulated gastric phase, the washed cell suspension was added to sterile simulated gastric fluid consisting of phosphate-buffered saline (PBS) adjusted to pH 2.5 and supplemented with pepsin at 3 mg/mL. The suspension was incubated at 37 °C for 3 h. After gastric exposure, aliquots were collected for viable cell enumeration. The remaining cell suspension was centrifuged and washed with sterile 0.85% (*w*/*v*) NaCl to remove residual gastric fluid before transfer to the intestinal phase.

#### 2.8.2. Tolerance to Simulated Intestinal Conditions

For the simulated intestinal phase, the gastric-exposed cells were resuspended in sterile simulated intestinal fluid consisting of PBS adjusted to pH 8.0, pancreatin at 3 mg/mL, and bile salts at 1% (*w*/*v*). The suspension was further incubated at 37 °C for 4 h. Viable cell counts were determined before exposure (T0), after the gastric phase (G3h), and after the intestinal phase (I4h).

#### 2.8.3. Control Group

The control group consisted of bacterial cells that were washed with 0.85% (*w*/*v*) NaCl and maintained in the same saline solution under identical incubation conditions as the experimental groups. These control samples served as a baseline reference for calculating the survival rates of the isolates.

#### 2.8.4. Enumeration of Viable Cells and Calculation Methods

##### Enumeration of Bacterial Cells (CFU/mL) Using the Drop Plate Method

Samples were serially diluted, and a 20 µL aliquot (0.02 mL) of each dilution was dropped onto agar plates and incubated until colonies were visible. Colonies were counted at dilutions yielding a countable range. The mean colony count ($\bar{x}$) of each dilution was calculated and used to determine the bacterial concentration (CFU/mL) according to the following equation:

$$\mathrm{CFU}/\mathrm{mL}=\bar{x}\times \frac{1}{V}\times \mathrm{dilution}\;\mathrm{factor}$$

where V is the volume plated (in mL). In this study, V = 0.02 mL; therefore, 1/V = 50. The equation can be expressed as:

$$\mathrm{CFU}/\mathrm{mL}=\bar{x}\times 50\times \mathrm{dilution}\;\mathrm{factor}$$

Note: The dilution factor refers to the reciprocal of the dilution used for plating (e.g., a dilution of 10^−4^ corresponds to a dilution factor of 10^4^).

##### Calculation of Survival Rate

Survival was recalculated from absolute viable counts (CFU/mL) to determine sequential survival (gastric, intestinal transition, and cumulative survival) and log reduction. The survival rate of the isolates after exposure to simulated gastrointestinal conditions was calculated by comparing the viable cell counts of the experiment group with those of the control group using the following equation:

$$\mathrm{Survival}\;\mathrm{rate}(\%)\;=\;\left(\frac{{\mathrm{CFU}/\mathrm{mL}}_{\mathrm{experiment}}}{{\mathrm{CFU}/\mathrm{mL}}_{\mathrm{control}}}\right)\times 100$$

#### 2.8.5. Selection Criteria for Isolates

Isolates showing a cumulative gastrointestinal log_10_ reduction of ≤1.0 were selected for further evaluation of additional probiotic-related properties.

### 2.9. Adhesion Ability Assay

Adhesion ability was assessed using a crystal violet staining method. After incubation, adherent cells were stained with 1% crystal violet, rinsed to remove excess dye, and destained with 95% ethanol. The OD of the destaining solution was measured at 570 nm. Experiments were performed in triplicate, and results were expressed as mean ± standard deviation (SD) [14,15,16].

This crystal violet assay measures biofilm-forming ability and abiotic surface attachment rather than specific adhesion. For each isolate, measurements were derived from nine technical replicate wells from a single experiment (blank replicate values were excluded). Corrected OD_570_ values were used as the primary indicator of attachment, whereas relative adhesion (%) is presented only for comparative purposes among isolates.

### 2.10. Molecular Identification by 16S rRNA Gene Sequencing

Selected *Bacillus* isolates were subjected to molecular identification via amplification of the 16S rRNA gene using polymerase chain reaction (PCR) with the universal primers 27F (5′-AGA GTT TGA TCC TGG CTC A-3′) and 1492R (5′-GGT TAC CTT GTT ACG ACT T-3′) [17]. The PCR products were sequenced, and the resulting sequences were compared with reference sequences in public databases using the EzBioCloud server [18] to determine species identity based on percentage similarity.

### 2.11. Whole-Genome Sequencing and Genome Analysis of Bacillus Subtilis

Bacterial genomic DNA was extracted and quantified using a Nanodrop Spectrophotometer (Thermo Fisher Scientific, Waltham, MA, USA) and a Qubit^®^ 4.0 Fluorometer (Invitrogen, Carlsbad, CA, USA). A ratio of ∼1.8 of absorbance at 260 and 280 nm and 2.0–2.2 of absorbance at 260 and 230 nm is accepted for further analysis. Quality-controlled DNA (total ~400 ng) was used for library preparation using a Rapid Sequencing gDNA—Barcoding (SQK-RBK114.24) Kit (ONT, Oxford, UK). The DNA library was prepared by transposase-mediated cleavage to produce chemically modified ends, followed by barcode addition to each DNA sample and final adapter ligation. The prepared library was then loaded into an R10.4.1 flow cell (FLO-MIN114; ONT, Oxford, UK) and sequenced using GridION with default settings. Dorado v0.9.0 (https://github.com/nanoporetech/dorado, accessed on 12 January 2025) using the super accuracy (SUP) model was used for base calling and quality control.

Raw Oxford Nanopore sequencing reads were initially assessed for sequencing quality using NanoPlot v.43.0 (https://github.com/wdecoster/NanoPlot, accessed on 12 January 2025) [19] to visualize read length distribution and base-calling quality metrics. Low-quality reads (Phred quality score < 15) and reads shorter than 1000 bp were subsequently filtered using NanoFilt v2.8.0 (https://github.com/wdecoster/nanofilt, accessed on 12 January 2025) [19]. De novo genome assembly of the filtered reads was performed using Flye v2.9.5 (https://github.com/fenderglass/Flye, accessed on 12 January 2025) [20], specifying an estimated genome size of 4 Mb. As its final step, Flye polished the draft assembly through read-based consensus correction to reduce residual base-calling errors. The quality of the assembled genome was evaluated using QUAST v5.2.0 (https://github.com/ablab/quast, accessed on 12 January 2025) [21], while genome completeness was assessed using BUSCO v5.8.2 (https://gitlab.com/ezlab/busco/-/releases/5.8.2, accessed on 12 January 2025) [22,23] based on lineage-specific single-copy orthologs. Potential genome contamination and completeness were further examined using CheckM v1.2.5 (https://github.com/Ecogenomics/CheckM, accessed on 12 January 2025) [23]. Gene prediction and functional annotation were performed using the Rapid Annotation using Subsystem Technology (RAST) server [24]. Taxonomic identification was conducted using the Type (Strain) Genome Server (TYGS) [25] by comparing the assembled genome against reference type strain genomes. Antimicrobial resistance (AMR) genes and potential virulence factors were identified using ABRicate version 1.2.0 (https://github.com/tseemann/abricate, accessed on 12 January 2025) by searching against four curated databases, including: (1) the NCBI AMRFinderPlus [26], (2) Comprehensive Antibiotic Resistance Database (CARD) [27], (3) ResFinder [28], and (4) the Virulence Factor Database (VFDB) [29]. Additionally, secondary metabolite biosynthetic gene clusters were identified using antiSMASH v8.0 [30].

### 2.12. IPEC-J2 Cell Culture

The intestinal porcine enterocyte cell line IPEC-J2 (derived from the jejunum of a neonatal, unsuckled piglet) was maintained in DMEM/F-12 (Gibco, Grand Island, NY, USA) supplemented with 10% (*v*/*v*) fetal bovine serum, 1% (*v*/*v*) antibiotic–antimycotic, and 1% (*v*/*v*) GlutaMAX at 37 °C in 5% CO_2_, with the medium replenished every 48 h. For adhesion assays, cells were seeded in 6-well plates at 3.5 × 10^5^ cells/well. For cytotoxicity, cells were seeded in 96-well plates at 1.0 × 10^4^ cells/well. All assays commenced when the monolayers reached 90–100% confluence.

### 2.13. In Vitro Adhesion Assay in IPEC-J2 Cells

The adhesion of *B. subtilis* 1BP1Co1 to IPEC-J2 cells was evaluated using confluent IPEC-J2 monolayers. Briefly, the monolayers were washed, pre-incubated in serum- and antibiotic-free DMEM/F-12, and then exposed to the bacterial suspension (1.0 × 10^9^ CFU/mL) for 2 h at 37 °C in 5% CO_2_. Non-adherent cells were removed by washing; adherent bacteria were released with 0.25% trypsin-EDTA, serially diluted, and plated on TSA. The assay was conducted in three independent experiments, each performed in duplicate. The adhesion rate was calculated using the following equation:

$$\mathrm{Adhesion}\;(\%)\;=\left(\frac{\mathrm{Adhered}\;\mathrm{bacteria}\;(\mathrm{CFU}/\mathrm{mL})}{\mathrm{Initial}\;\mathrm{added}\;\mathrm{bacteria}\;(\mathrm{CFU}/\mathrm{mL})}\right)\times 100$$

### 2.14. In Vitro Cytotoxicity Assay (MTT)

IPEC-J2 viability after exposure to *B. subtilis* 1BP1Co1 was determined using an MTT assay. Confluent monolayers in 96-well plates were inoculated with washed bacterial suspensions at final concentrations ranging from 1.0 × 10^4^ to 1.0 × 10^7^ CFU/mL, corresponding to nominal multiplicities of infection (MOI) of 1:1, 1:10, 1:100 and 1:1000 based on the initial seeding density. Untreated cells served as the 100% viability control, whereas cells treated with 25% (*v*/*v*) DMSO served as the cell-death control. After 6 h of incubation at 37 °C, MTT (0.5 mg/mL) was added to each well for 3 h. The resulting formazan crystal was solubilized in DMSO, and the absorbance was measured at 595 nm.

### 2.15. Statistical Analysis

Inhibition-zone diameters were analyzed using the Kruskal–Wallis test followed by Dunn’s multiple comparison test, as zone data are typically non-normally distributed and include non-inhibited (0 mm) values. The gastrointestinal tolerance assay was performed with three technical replicate drops per dilution; viable counts are expressed as log_10_ CFU/mL (mean ± SD). Biofilm and adhesion data are presented as mean OD_570_ ± SD. Cell viability (MTT) data were analyzed by one-way ANOVA followed by Tukey’s post hoc test. All statistical analyses were performed using IBM SPSS Statistics v29; *p* < 0.05 was considered statistically significant.

## 3. Results

### 3.1. Isolation and Distribution of Bacillus spp.

A total of 59 *Bacillus* isolates were obtained across all sampled anatomical sites. The highest number of isolates was recovered from the cecum, followed by the ileum and feces. The distribution of isolates from each gastrointestinal section is presented in Table S1.

### 3.2. Colony and Cellular Morphology

The isolates exhibited diverse colony morphologies, including circular, flat, wrinkled, and irregular forms with smooth or rough surfaces. Microscopic observation revealed rod-shaped cells arranged in chains or pairs, with several isolates forming endospores.

### 3.3. Hemolytic Activity

Hemolytic activity varied among the isolates, with γ-hemolysis being the most common pattern. Isolates exhibiting β-hemolysis were excluded from subsequent analyses (Table S2).

### 3.4. Antimicrobial Activity

**Figure 2:**
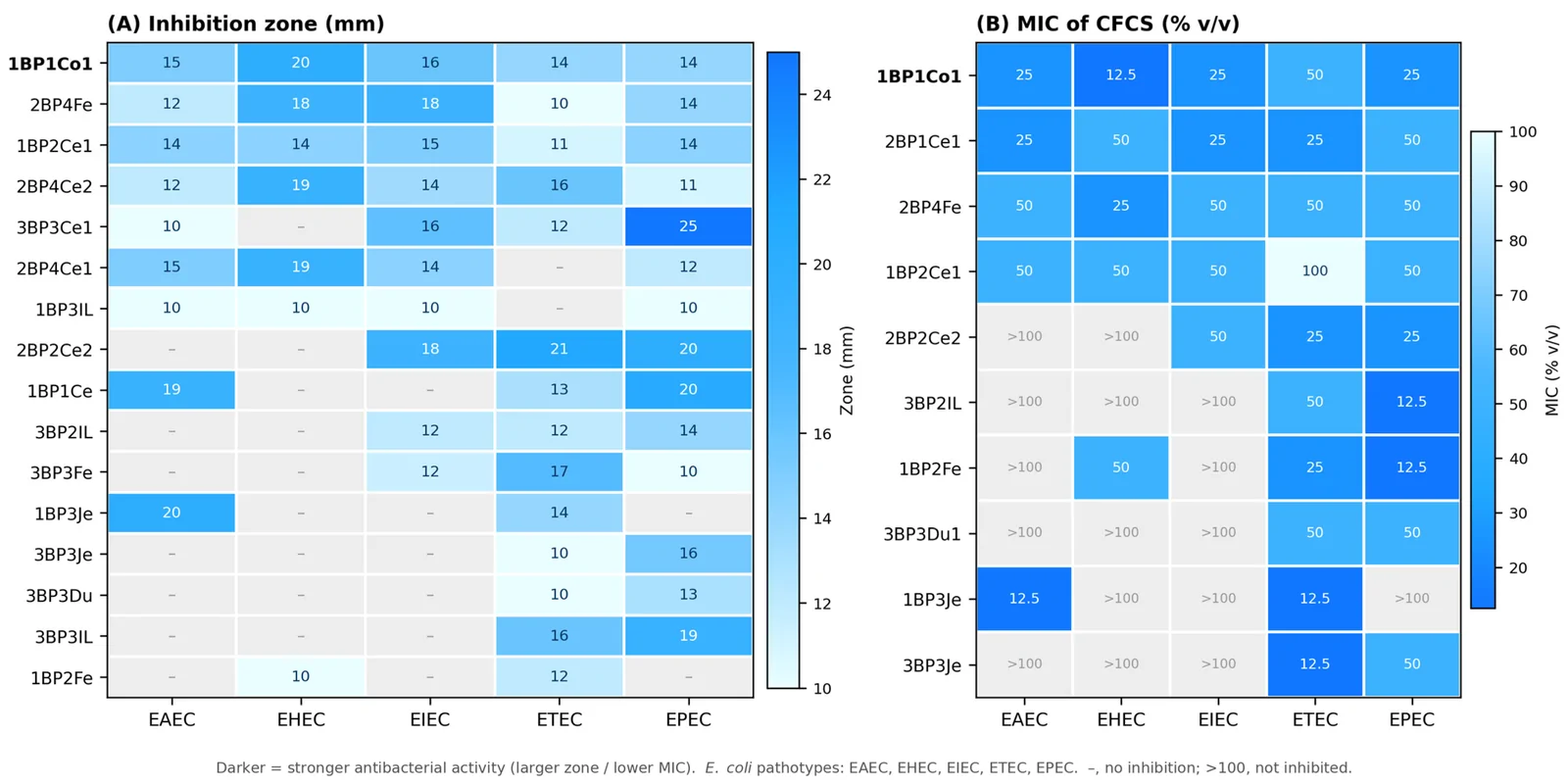
Antibacterial activity of *Bacillus* isolates against five diarrheagenic *E. coli* pathotypes. (**A**) Inhibition-zone diameters (mm) from the agar slab assay; (**B**) minimum inhibitory concentrations (% *v*/*v*) of cell-free culture supernatants. Darker shading indicates stronger activity (larger zone or lower MIC). Candidate 1BP1Co1 was the only isolate active against all five pathotypes. “–“, no inhibition; “>100”, not inhibited at the highest concentration tested.

The primary screening via the agar slab assay revealed that several Bacillus isolates successfully inhibited at least one pathogenic *E. coli* pathotype (Figure 2). While inhibition-zone diameters varied, isolate selection was based on the overall antibacterial profile rather than the maximum zone diameter alone. Isolate 1BP1Co1 was ultimately selected for further characterization because it demonstrated the most robust and consistent broad-spectrum inhibition across multiple pathogenic *E. coli* pathotypes [31,32].

The antibacterial activity of the selected isolate was quantitatively confirmed by determining the MIC of its CFCS. The CFCS of *Bacillus* isolate 1BP1Co1 exhibited strong inhibitory activity, with MIC values ranging from 12.5% to 50% (*v*/*v*) against the tested *E. coli* pathotypes (Table S3).

#### Minimum Inhibitory Concentration of Cell-Free Culture Supernatants

The MIC results confirmed the antibacterial activity of isolate 1BP1Co1 against pathogenic *E. coli*, supporting its selection for subsequent genomic characterization [33,34]. The CFCS from 1BP1Co1 inhibited all five pathogenic *E. coli* pathotypes (EAEC, EHEC, EIEC, ETEC, and EPEC) with MIC values of 25%, 12.5%, 25%, 50%, and 25% (*v*/*v*), respectively. These results indicate that 1BP1Co1 exhibited both a broad inhibitory spectrum and a favorable overall MIC profile compared with the other isolates showing broad activity. Notably, the MIC against EHEC was 12.5% (*v*/*v*), which was the lowest MIC observed for this isolate among the tested pathogenic groups.

While isolates 2BP1Ce1, 1BP2Ce1, and 2BP4Fe also inhibited all five pathotypes, their overall MIC profiles were less potent than that of 1BP1Co1. Specifically, MIC values ranged from 25% to 50% (*v*/*v*) for both 2BP1Ce1 and 2BP4Fe, and from 50% to 100% (*v*/*v*) for 1BP2Ce1. Conversely, isolates 2BP2Ce2 and 1BP2Fe exhibited strong inhibitory activity against specific pathotypes but failed to inhibit all five within the highest CFCS concentrations tested, indicating a narrower inhibitory spectrum.

The complete OD_600_ values used to support the visual MIC interpretation are provided in Table S3. As expected, higher CFCS concentrations correlated with lower OD_600_ values relative to the growth controls, confirming a dose-dependent reduction in bacterial growth. Taken together, the combined quantitative data from the agar slab and MIC assays identified 1BP1Co1 as the most promising broad-spectrum antibacterial candidate, justifying its selection for further genomic and probiotic evaluations.

### 3.5. Tolerance to Simulated Gastrointestinal Conditions

**Figure 3:**
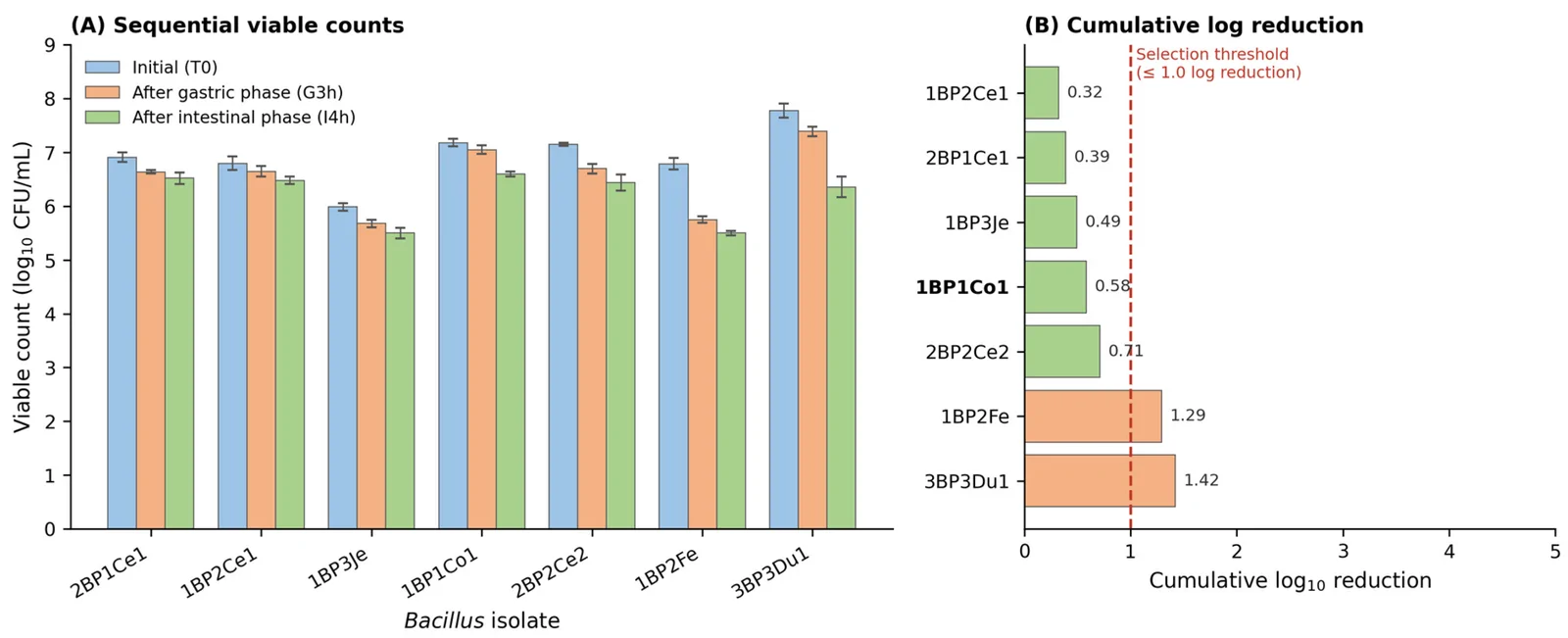
Survival of selected *Bacillus* isolates under sequential simulated gastrointestinal conditions. (**A**) Viable counts (log_10_ CFU/mL) at baseline (T0), after 3 h in simulated gastric fluid (G3h) and after a further 4 h in simulated intestinal fluid (I4h). (**B**) Cumulative log_10_ reduction; the dashed line marks the ≤1.0-log selection threshold.

The *Bacillus* isolates exhibited strain-dependent responses to the sequential simulated gastrointestinal stress (Figure 3, Table 1). After 3 h of exposure to simulated gastric fluid (pH 2.5 supplemented with pepsin), survival varied widely; several isolates maintained high viable counts, whereas others showed a marked decrease in viability. Following subsequent transfer to simulated intestinal fluid (containing pancreatin and bile salts) for 4 h, further viability reductions were observed in several strains.

Among the tested candidates, isolate 1BP1Co1 demonstrated robust and consistent tolerance throughout the entire sequential exposure model. It maintained high viable cell counts after the gastric phase and retained robust viability following the intestinal phase, yielding a cumulative log_10_ reduction of 0.58 and a cumulative gastrointestinal survival rate of 26.30%. These results indicate that 1BP1Co1 is highly resilient to standard gastrointestinal stress conditions.

### 3.6. Adhesion Ability

The adhesion ability of 40 *Bacillus* spp. isolates, determined by crystal violet staining and expressed as OD_570_ values, is presented in Table 2. Overall, the OD_570_ values varied among the isolates, indicating differences in adhesion capacity, with measured values ranging from 0.097 ± 0.008 to 1.706 ± 0.002.

Among the cecal isolates, the mean OD_570_ values ranged from 0.112 ± 0.009 to 1.572 ± 0.002. Mid-colon isolates showed OD_570_ values ranging from 0.154 ± 0.003 to 1.706 ± 0.002, whereas ileal isolates ranged from 0.115 ± 0.002 to 1.482 ± 0.006. Fecal isolates exhibited OD_570_ values between 0.097 ± 0.008 and 1.373 ± 0.002. Duodenal isolates ranged from 0.114 ± 0.012 to 1.538 ± 0.003, while jejunal isolates showed values from 0.118 ± 0.011 to 1.482 ± 0.002. Across all isolates tested, 1BP1Co1 exhibited the highest adhesion ability (1.706 ± 0.002), followed by 1BP2Ce1 (1.572 ± 0.002), 3BP3Du1 (1.538 ± 0.003), 3BP2IL (1.482 ± 0.006), and 3BP3Je (1.482 ± 0.002).

Following biofilm and abiotic-surface screening, candidate *B. subtilis* 1BP1Co1 was validated on the IPEC-J2 porcine jejunal epithelial cell line. The strain attached to differentiated IPEC-J2 monolayers with an adhesion efficiency of 0.14 ± 0.035%.

In the MTT assay, IPEC-J2 viability remained above 90% at all bacterial densities tested (91.9 ± 6.17% to 98.4 ± 12.27%; MOI 1:1 to 1:1000), with no significant difference from the untreated control, whereas the 25% (*v*/*v*) DMSO control reduced viability to 58.8 ± 5.94% (*p* < 0.01). These results indicate that 1BP1Co1 attaches to porcine intestinal epithelium and is non-cytotoxic in vitro (Figure S1).

### 3.7. Molecular Identification

Based on the overall screening results obtained from the phenotypic evaluations, isolate 1BP1Co1 was selected as a representative probiotic candidate for whole-genome sequencing. The purpose of genome analysis was to confirm its taxonomic identity and to evaluate its genomic safety and functional potential, rather than to identify the phenotypically highest-performing isolate from a single assay.

The 16S rRNA gene sequencing confirmed that the selected isolates belonged to the genus *Bacillus* within the *Bacillus subtilis* species complex (sequence similarity 92.25–100%; Table S4). Because members of this complex share very high 16S rRNA identity, this marker did not provide reliable species-level resolution [35]; definitive identification of the candidate isolate 1BP1Co1 was therefore performed via whole-genome analysis.

### 3.8. Genome Assembly and Annotation of Isolate 1BP1Co1

Oxford Nanopore sequencing generated a total of 292,898 reads, yielding 1.02 Gb of data, with a mean read length of 3488 bp (N50 = 7611 bp) and a median read quality of Q14.1. The assembled genome comprised a single circular chromosome of 4,148,036 bp with a GC content of 43.65% and an average sequencing coverage of 242X. The assembly was highly contiguous, consisting of one contig with an N50 equal to the genome size, and exhibited high completeness (99.10%) with minimal contamination (0.16%). Genome annotation from RAST predicted 4275 protein-coding sequences, along with 30 rRNA genes and 86 tRNA genes. The genome assembly and annotation statistics of the 1BP1Co1 isolate are summarized in Table 3.

### 3.9. Taxonomic Identification of Isolate 1BP1Co1

**Figure 4:**
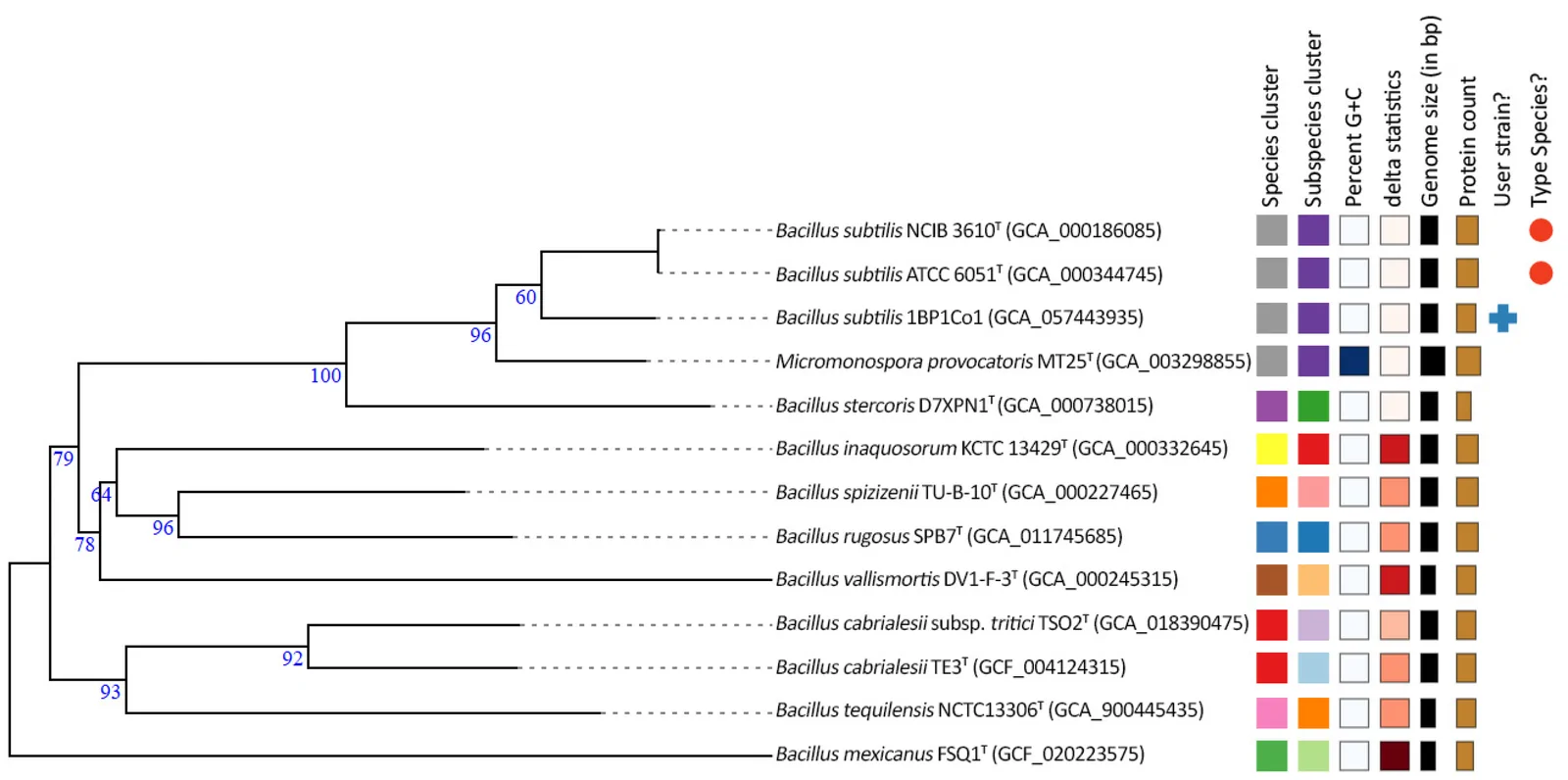
Phylogenomic tree of the 1BP1Co1 isolate and related *Bacillus* species based on whole-genome sequence analysis.

Taxonomic classification of the 1BP1Co1 isolate was performed using the TYGS, integrating digital DNA–DNA hybridization (dDDH) and phylogenomic analysis. The dDDH results demonstrated high genomic similarity between the isolate and *Bacillus subtilis* reference strains, with values of 93.8% and 92.4% against *Bacillus subtilis* ATCC 6051 and NCIB 3610, respectively, exceeding the accepted species delineation threshold of 70%. Consistent with these findings, phylogenomic analysis placed the 1BP1Co1 isolate within the *Bacillus subtilis* clade, clustering with the type strains NCIB 3610^T^ and ATCC 6051^T^ (GBDP pseudo-bootstrap support of 60; shown in Figure 4). Although relatively high dDDH values were also observed with closely related members of the *Bacillus subtilis* species complex, including *Bacillus stercoris* (90.5%), *Bacillus cabrialesii* (87.6%), and *Bacillus spizizenii* (86.8%), the isolate was clearly resolved within the *Bacillus subtilis* lineage. In contrast, more distantly related taxa such as *Bacillus tequilensis*, *Bacillus vallismortis*, and *Bacillus mexicanus* showed lower dDDH values (~66–68%) and formed separate branches in the phylogenomic tree, while *Micromonospora provocatoris* was positioned as a distant outgroup (13.1%). Collectively, these results provide strong genomic evidence supporting the taxonomic assignment of the 1BP1Co1 isolate to *Bacillus subtilis*, with dDDH analysis summarized in Table S5 and the phylogenomic tree presented in Figure 4.

### 3.10. Identification of AMR and Virulence Genes

Screening the assembled genome against multiple databases revealed the presence of several resistance-associated genes (Table S6). A total of 11 AMR genes were identified using the CARD, while 4 and 2 genes were detected using the NCBI AMR database and ResFinder, respectively. The AMR genes detected in the 1BP1Co1 isolate were primarily associated with intrinsic resistance mechanisms, including multidrug efflux pumps (e.g., *blt*, *bmr*, *ykkC*, and *ykkD*) and ribosomal protection mediated by an ABC-F protein (*vmlR*), which are commonly found in environmental *Bacillus* species. Additionally, a limited number of genes related to specific antibiotic resistance, such as *aadK* (aminoglycoside resistance) and *mphK* (macrolide resistance), were identified. However, no clinically relevant high-risk AMR genes were detected, suggesting a low antimicrobial resistance risk profile for this strain. In parallel, virulence factor screening using the VFDB revealed the complete absence of known virulence factor genes, indicating that the strain likely lacks established virulence-associated determinants and may therefore have low pathogenic potential.

### 3.11. Analysis of Secondary Metabolite Production Genes

The capacity to synthesize antimicrobial secondary metabolites is a well-characterized trait among various *Bacillus* species, particularly within potential probiotic strains like the commercially available *Bacillus subtilis* DE111^®^ and MTCC 5981 [36,37]. To further investigate this, we analyzed the secondary metabolite biosynthetic gene clusters (BGCs) within the completely assembled genome (scaffold_1) of *Bacillus* isolate 1BP1Co1 using antiSMASH v8.0.4 [30]. Consequently, a total of 11 BGC regions were identified on the primary contig (scaffold_1) (Table 4). Among these, KnownClusterBlast analysis revealed that six regions shared high similarity with known reference clusters in the MIBiG database (v.3.1), specifically: Region 2 (BGC0001089; bacillaene), Region 3 (BGC0001095; fengycin), Region 7 (BGC0000309; bacillibactin), Region 8 (BGC0000602; subtilosin A), Region 9 (BGC0001184; bacilysin), and Region 10 (BGC0000433; surfactin).

The predicted bioactivity function of these secondary metabolites produced from BGCs found in *Bacillus* isolate 1BP1Co1 was shown in Table 4. Briefly, *Bacillus* isolate 1BP1Co1 conferred BGCs for synthesizing several important secondary metabolites that have been reported throughout the commercially important species of *Bacillus subtilis* and *Bacillus licheniformis* [38]. The predicted biological activities of the secondary metabolites derived from the BGCs in *Bacillus* isolate 1BP1Co1 were summarized in Table 5. Overall, isolate 1BP1Co1 harbors BGCs responsible for the synthesis of several key secondary metabolites previously reported in commercially valuable species such as *Bacillus subtilis* and *Bacillus licheniformis* [39,40,41]. BGCs encoding non-ribosomal peptide (NRP) secondary metabolites included those for fengycin (BGC0001095, located in Region 3), bacillibactin (BGC0000309, located in Region 7), bacilysin (BGC0001184, located in Region 9), and surfactin (BGC0000433, located in Region 10). The biosynthesis of these NRPs requires the activity of non-ribosomal peptide synthetases (NRPS) [39]. Notably, these peptides display broad-spectrum antimicrobial activity against various pathogens. Specifically, fengycin is active against *Staphylococcus aureus* and *Listeria monocytogenes*; surfactin is effective against *Klebsiella pneumoniae*, *Pseudomonas aeruginosa*, and *S. aureus*; and bacilysin showed activity against *E. coli*. [40,41,42,43].

Furthermore, isolate 1BP1Co1 was found to harbor a BGC responsible for the synthesis of the polyketide bacillaene (BGC0001089, located in Region 2). The biosynthesis of bacillaene requires the hybrid activity of NRPS and type I polyketide synthase (PKS) complexes. Bacillaene exhibits significant antimicrobial activity against several strains of *Staphylococcus aureus* and *E. coli* [44]. Furthermore, the genome of isolate 1BP1Co1 contains a BGC encoding a ribosomal peptide, specifically subtilosin A (BGC0000602, located in Region 8). Subtilosin A, a class I bacteriocin initially discovered in *Bacillus subtilis* 168, is a well-demonstrated antimicrobial peptide with broad-spectrum activity against both Gram-positive and Gram-negative bacteria [45].

**Table 5:** Secondary metabolites predicted in *Bacillus* isolate 1BP1Co1 and their reported bioactivities.

Region	Secondary Metabolites	Chemical Structure	Reported Biological Activity	References
2	Bacillaene	Polyketides	Bacteriostatic antibiotic, inhibiting prokaryotic protein synthesis; broad-spectrum against several bacteria	[44,46,47]
3	Fengycin	Cyclic lipopeptide	Biosurfactant antifungal; reducing expression of virulence factors and biofilm formation in *S. aureus*	[41,48]
7	Bacillibactin	Siderophores	Obtaining iron from environment	[49]
8	Subtilosin A	Cyclic peptides	Antimicrobial activity against a wide range of Gram-positive and Gram-negative bacteria	[50]
9	Bacilysin	Dipeptide	Antimicrobial activity against a wide range of bacteria; antimicrobial activity against *Candida albicans*	[43]
10	Surfactin	Lipopeptide	Biosurfactant; inhibitory activity against a wide range of bacteria; cell wall and plasma membrane rupture damage in inhibition against *S. aureus*	[42,51,52]

## 4. Discussion

The present study aimed to isolate and characterize *Bacillus* spp. from different sections of the gastrointestinal tract of healthy pigs and to evaluate their probiotic potential based on several key criteria. The results demonstrated that several isolates possessed desirable probiotic characteristics, suggesting their potential application as probiotic candidates for improving gut health and controlling enteric pathogens in livestock.

The gastrointestinal tract of animals represents an important reservoir of diverse microbial communities, including spore-forming bacteria belonging to the genus *Bacillus*. In this study, a total of 59 *Bacillus* isolates were obtained from different sections of the pig gastrointestinal tract, with a higher proportion isolated from the cecum and ileum. Similar findings have been reported in previous studies where *Bacillus* spp. were isolated from poultry and livestock intestinal tracts, suggesting that host-associated strains are well adapted to intestinal conditions [1,53]. Host-derived probiotic strains are often considered more suitable for probiotic development because they are naturally adapted to survive and function within the host intestinal environment [54]. Although Bacillus spp. are generally regarded as facultative or transient members of the porcine gut microbiota rather than dominant resident bacteria, their presence and interactions within the intestinal tract have been documented in several studies. Recent evidence suggests that *B. subtilis* can contribute to gut microbial balance, intestinal health, and host performance in pigs [11,55]. These observations indicate that *Bacillus* spp. is naturally associated with the porcine gastrointestinal ecosystem and may therefore represent a valuable source of host-associated probiotic candidates. Consequently, the isolation of *B. subtilis* 1BP1Co1 from healthy pigs provides additional biological rationale for its evaluation as a potential probiotic strain for swine production.

Morphological and microscopic examinations confirmed that the isolates exhibited typical characteristics of *Bacillus* spp., including rod-shaped cells and the ability to form endospores. Spore formation is a critical trait that contributes to the high stability and survivability of *Bacillus* probiotics during feed processing and gastrointestinal passage [56]. Unlike many non-spore-forming probiotic bacteria, *Bacillus* spores can survive harsh environmental conditions such as heat, desiccation, and acidic pH, allowing them to reach the intestine in a viable form where they can germinate and exert beneficial effects [57].

The practical application of *Bacillus* probiotics in commercial feed production is strongly influenced by their ability to withstand feed-processing conditions. Previous studies reported that spores of *Bacillus licheniformis* remained viable after exposure to temperatures ranging from 80 to 120 °C and successfully survived the extrusion process during aquafeed production [58]. Probiotic formulations containing *B. subtilis* remained effective following feed pelleting at temperatures ranging from 75 to 90 °C, with probiotic supplementation improving feed conversion efficiency and enhancing intestinal secretory IgA responses in broilers regardless of the pelleting temperature applied [59]. These findings suggest that *Bacillus* spores can maintain their biological functionality after exposure to common feed manufacturing processes. Nevertheless, although the present study confirmed the spore-forming capability of *B. subtilis* 1BP1Co1, the survival rate of *B. subtilis* 1BP1Co1 spores under different feed granulation temperatures and humidity conditions was not experimentally evaluated. Therefore, further studies under simulated commercial feed-processing and storage conditions are warranted to validate the industrial applicability of this strain as a probiotic feed additive.

Safety assessment is a fundamental requirement for probiotic selection. In the present study, hemolytic activity tests revealed that the majority of isolates exhibited γ-hemolysis, indicating the absence of hemolytic activity. This finding suggests that these isolates are unlikely to possess pathogenic traits. Similar safety screening approaches have been applied in previous studies evaluating probiotic *Bacillus* spp., where non-hemolytic isolates were selected as potential probiotic candidates [8,60]. The absence of hemolytic activity is widely used as an initial indicator of probiotic safety [54].

Another essential characteristic of probiotic microorganisms is their ability to survive under harsh gastrointestinal conditions. In this study, selected isolates demonstrated high survival rates under simulated gastric and intestinal conditions, including exposure to acidic pH and bile salts. These results indicate that the isolates possess strong tolerance to gastrointestinal stress conditions. Similar findings have been reported for several *Bacillus subtilis* strains isolated from animals, which showed high resistance to acidic environments and bile salts, enabling them to survive gastrointestinal transit [2,8]. Such tolerance is essential for probiotic bacteria to reach the intestine and colonize the host gut [61].

Adhesion ability is another important property of probiotic microorganisms because it facilitates colonization of the intestinal mucosa and prolongs their persistence in the gastrointestinal tract. In this study, adhesion assays demonstrated variability among isolates, with some strains showing relatively strong adhesion ability. Adhesion capacity is often associated with bacterial surface proteins, extracellular polysaccharides, and biofilm-forming ability, which contribute to bacterial attachment to intestinal epithelial cells [5]. Similar adhesion properties have been reported for *B. subtilis* AJQ03, which demonstrated strong adhesion to intestinal mucus and effectively inhibited pathogen colonization [8].

One of the most significant findings of this study was the antimicrobial activity exhibited by several isolates against pathogenic *E. coli* strains, including EAEC, EHEC, EIEC, ETEC, and EPEC. Some isolates showed strong inhibitory activity, producing large inhibition zones against these pathogens. The ability of *Bacillus* spp. to inhibit pathogenic bacteria may be attributed to multiple inhibitory mechanisms. *B. subtilis* strains are known to produce various antimicrobial metabolites, including lipopeptides such as surfactin, fengycin, and bacillomycin, as well as bacteriocins and other secondary metabolites [7,9]. These compounds play an important role in suppressing the growth of pathogenic microorganisms in the intestinal environment. One of the primary mechanisms involves the production of antimicrobial metabolites that disrupt bacterial cell membranes and interfere with essential cellular processes. These bioactive compounds can increase membrane permeability, leading to leakage of intracellular contents and ultimately causing cell death [7]. In addition, *Bacillus* probiotics can inhibit pathogenic bacteria through competitive exclusion, whereby probiotic strains compete with pathogens for adhesion sites and nutrients in the intestinal environment. This competition limits the ability of pathogenic *E. coli* to colonize the intestinal mucosa, thereby reducing infection and disease risk in the host [54]. The combination of antimicrobial compound production and competitive exclusion likely contributes to the strong inhibitory activity observed against multiple *E. coli* pathotypes in the present study.

The inhibition of pathogenic *E. coli* is particularly important in swine production because it is one of the primary causes of post-weaning diarrhea in piglets. Several studies have demonstrated that supplementation with *B. subtilis* probiotics can significantly reduce intestinal populations of *E. coli* while improving growth performance and gut health in pigs [2,11]. Similarly, dietary supplementation with *B. subtilis* has been reported to alleviate diarrhea and enhance immune function in weaned pigs [5].

In addition to antimicrobial activity, probiotic *Bacillus* spp. may contribute to host health through the modulation of intestinal microbiota and the enhancement of digestive functions. Previous studies have shown that *B. subtilis* supplementation can improve digestive enzyme activity, enhance intestinal morphology, and regulate gut microbiota composition in piglets and poultry [11,62]. Furthermore, probiotics can improve intestinal barrier integrity and immune responses, thereby reducing susceptibility to intestinal infections [5,63].

Molecular identification based on 16S rRNA gene sequencing revealed that several isolates were closely related to *B. siamensis* and *B. velezensis*, which belong to the *B. subtilis* species complex. Because the highly conserved nature of the 16S rRNA gene precludes species-level resolution within this complex [35], the definitive identification of the candidate isolate 1BP1Co1 as *B. subtilis* was obtained from whole-genome analysis (TYGS and dDDH). Members of this complex are known for their strong antimicrobial properties and production of bioactive metabolites. Recent genomic studies have demonstrated that these species possess BGCs responsible for the biosynthesis of antimicrobial compounds, highlighting their potential for probiotic and biocontrol applications [7,9].

The collective BGCs identified in isolate 1BP1Co1 (Table 4 and Table 5) indicated the presence of multiple regions dedicated to antimicrobial secondary metabolite production, which is a key identified characteristic of spore-forming probiotics, including *B. subtilis* and *B. licheniformis* [37]. In particular, these bioactive secondary metabolites physiologically protect the viability and competitiveness of these spore-forming probiotics during sporulation, which is likely to occur during the stationary phase of cell growth [64]. Mechanistically, bacillaene has been shown to selectively inhibit bacterial protein biosynthesis [44]. Furthermore, both surfactin and fengycin function as biosurfactants that target the bacterial envelope. Specifically, fengycin has been shown to cause cell membrane damage upon binding [65]. In contrast, surfactin strongly disrupts membrane permeability by forming channels. Additionally, upon penetrating the lipid bilayer, surfactin alters membrane thickness and disrupts hydrocarbon arrangement [66]. A recent study by Yan et al. (2024) also demonstrated that the co-production of surfactin and fengycin by *B. subtilis* BBW1542 holds great promise as a biocontrol agent against various foodborne pathogens [67]. Subtilosin A, on the other hand, exerts antibacterial activity by disrupting the transmembrane pH gradient upon binding to the membrane receptor, eventually leading to ATP efflux and subsequent cell death [68]. Furthermore, subtilosin A has been shown to disrupt intercellular quorum sensing, thereby preventing biofilm formation [69]. Bacillibactin is known as an iron chelating agent that exerts antimicrobial activity by reducing iron bioavailability for competing microorganisms [64,70]. In conclusion, the presence of these secondary metabolite BGCs in isolate 1BP1Co1 highlighted the probiotic potential of this newly isolated *Bacillus* strain to mitigate enteric *E. coli*-associated post-weaning diarrhea in swine production systems. Future studies employing transcriptomic and metabolomic approaches are essential to elucidate the detailed mechanisms of the production of these antimicrobial secondary metabolites in this newly isolated *Bacillus* sp. Overall, the findings of the present study demonstrate that several *Bacillus* isolates obtained from the gastrointestinal tract of pigs exhibit multiple desirable probiotic properties, including safety, tolerance to gastrointestinal stress, adhesion ability, and antimicrobial activity against pathogenic *E. coli*. These characteristics suggest that the selected isolates may serve as promising probiotic candidates for improving gut health and controlling enteric pathogens in swine production.

However, further studies are required to confirm the probiotic efficacy of these isolates under in vivo conditions. Future research should include animal feeding trials, detailed genomic safety assessments, and evaluation of their effects on intestinal microbiota and host immune responses. Such investigations will provide more comprehensive evidence supporting the potential application of these isolates as probiotic feed additives in livestock production [71,72].

The genomic study provides comprehensive genomic insights into the *B. subtilis* 1BP1Co1 isolate, highlighting its potential as a safe and functional probiotic candidate. The high-quality genome assembly, characterized by a single circular chromosome with high completeness (99.10%) and minimal contamination (0.16%), confirms the robustness of the sequencing and assembly pipeline, consistent with previous studies utilizing long-read sequencing technologies for *Bacillus* genomes [20,23]. The genome size (~4.15 Mb) and GC content (43.65%) are in agreement with typical *B. subtilis* genomes, supporting accurate taxonomic classification [25].

Phylogenomic analysis and dDDH values (>90%) clearly placed the 1BP1Co1 isolate within the *B. subtilis* species, closely related to reference strains such as ATCC 6051 and NCIB 3610. This taxonomic positioning is important, as *B. subtilis* is widely recognized as a Generally Regarded as Safe (GRAS) microorganism with extensive applications in probiotics and functional feed additives [56,57]. Members of this species are known for their resilience, including spore-forming capability, which enhances survival under gastrointestinal conditions [54].

A key finding of this study is the identification of biosynthetic potential linked to antimicrobial activity, particularly the presence of genes associated with lipopeptide production. Among these, the *srfA* gene cluster, responsible for surfactin biosynthesis, is of particular importance. Surfactin is a powerful cyclic lipopeptide biosurfactant encoded by the *srfA* operon (*srfAA, srfAB, srfAC*, and *srfAD*) [73]. While it exhibits minimal direct toxicity against most Gram-negative bacteria, its potent biosurfactant properties act synergistically to compromise the outer membrane lipid bilayer of Gram-negative bacteria such as *E. coli*, thereby facilitating the penetration of other antimicrobial agents [74]. In contrast, surfactin demonstrates strong bactericidal activity against Gram-positive bacteria, including *Staphylococcus aureus* and *Listeria monocytogenes*, where it can directly intercalate into the cytoplasmic membrane, leading to membrane solubilization and cell lysis [64]. This differential activity highlights the dual functional role of surfactin as both a direct antimicrobial agent and a membrane-permeabilizing facilitator, contributing to a synergistic mode of action against a broad spectrum of pathogens.

The presence of the *srfA* gene cluster in the 1BP1Co1 genome suggests a strong intrinsic capacity for antimicrobial metabolite production, which may explain the observed probiotic potential of *Bacillus* isolates reported in previous studies [8,9]. In addition to direct antimicrobial effects, surfactin has been shown to modulate host immune responses and enhance intestinal barrier integrity, further supporting its role in probiotic efficacy [7]. This multifunctional activity positions the *srfA* gene as a critical molecular marker for evaluating probiotic candidates within the *Bacillus* genus. Although this present study verified the relationship between predicted secondary metabolite BGCs and antimicrobial activity, the safety of key lipopeptides, especially surfactin, toward mammalian and intestinal cells has been well-documented. Wang et al. verified the cytotoxicity effect on porcine intestinal epithelial cells (IPEC-J2) using an MTT assay and found that the safe dose of surfactin was up to 0.02 mg/mL. Moreover, using surfactin at low concentration can prevent the infection of transmissible gastroenteritis virus [75]. Consistently, endospore suspension of surfactin-producing *Bacillus* strains showed no adverse effect on intestinal enterocytes (Caco-2) with cell viability > 80% [76].

Importantly, the genomic safety assessment revealed a low-risk profile for the 1BP1Co1 isolate. Although several AMR genes were identified, these were primarily associated with intrinsic resistance mechanisms such as efflux pumps (e.g., *blt*, *bmr*, *ykkC*, and *ykkD*) and ribosomal protection proteins (*vmlR*), which are commonly found in environmental *Bacillus* spp. and are not typically linked to horizontal gene transfer or clinical risk [26,27]. The absence of high-risk or acquired AMR genes further supports the safety of this strain for potential applications. Moreover, the lack of detectable virulence factor genes, as confirmed using the VFDB, reinforces the non-pathogenic nature of the isolate [29]. This finding is consistent with previous reports indicating that probiotic *B. subtilis* strains generally lack classical virulence determinants and exhibit low pathogenic potential [57,60]. Together, these results suggest that the 1BP1Co1 isolate meets key safety criteria required for probiotic development.

In the context of probiotic functionality, *B. subtilis* strains have been widely reported to improve host growth performance, enhance immune responses, and modulate gut microbiota composition in both livestock and aquaculture systems [2,5,62]. The genomic features identified in this study, particularly the presence of the *srfA* gene cluster, further support the potential of the 1BP1Co1 isolation to exert beneficial effects through antimicrobial activity and host interaction. From an industrial application perspective, *Bacillus* spore-formers are routinely incorporated into commercial swine and poultry diets together with organic acids and exogenous enzymes. A previous study demonstrated that *Bacillus*-based biotics and enzyme cocktail benefit nursery pigs in terms of reducing pathogenic *Mycoplasma* spp. and improving immunity [77]. Additionally, it was found that supplementation of *Bacillus* probiotic (*Bacillus coagulans*) with benzoic acid could improve growth performance and intestinal barrier integrity in pigs fed an antibiotic-free diet [78].

Overall, this study demonstrates that the *B. subtilis* 1BP1Co1 isolate possesses a combination of desirable probiotic traits, including genomic stability, antimicrobial biosynthetic potential, and a favorable safety profile. Future studies should focus on functional validation of surfactin production, gene expression analysis under gastrointestinal conditions, and in vivo evaluation of probiotic efficacy to fully establish its application potential in food and feed industries.

## 5. Conclusions

This study successfully isolated and characterized host-associated *Bacillus* species from the gastrointestinal tracts of healthy pigs, demonstrating their potential as sustainable probiotic alternatives to mitigate enteric disorders like post-weaning diarrhea. Through comprehensive phenotypic screening, several isolates exhibited essential probiotic characteristics, including the absence of hemolytic activity, robust tolerance to simulated gastrointestinal stress such as acidic pH and bile salts, and notable mucosal adhesion capabilities. Importantly, these selected isolates demonstrated broad-spectrum inhibitory activity in vitro against pathogenic *E. coli,* which are primary etiological agents of enteric infections in swine.

Advanced genomic analysis of the highly promising isolate, *B. subtilis* 1BP1Co1, further validated its suitability and safety for probiotic development. Whole-genome sequencing confirmed a highly safe genomic profile, characterized by the complete absence of known virulence factors and clinically relevant, high-risk antimicrobial resistance genes. Furthermore, genome annotation elucidated the molecular mechanisms underlying the strain’s pathogen-inhibitory potential by identifying multiple biosynthetic gene clusters dedicated to the production of potent secondary metabolites. These included genomic regions responsible for synthesizing non-ribosomal peptides and polyketides such as surfactin, fengycin, bacillaene, and subtilosin A, which collectively act to disrupt bacterial cell membranes and inhibit vital cellular processes in competing pathogens.

Collectively, these phenotypic and genomic findings position indigenous *Bacillus* strains, particularly *B. subtilis* 1BP1Co1, as promising probiotic candidates with the potential to be developed for controlling enteric infections in swine production systems. To advance these candidates toward commercial and industrial application, future research must prioritize in vivo animal feeding trials to confirm their efficacy within the host, along with transcriptomic and metabolomic approaches to fully elucidate the dynamic mechanisms of antimicrobial secondary metabolite production within the intestinal environment.

## Figures and Tables

**Table 1:** The results of the tolerance test of *Bacillus* spp. to simulated gastrointestinal conditions.

Isolate	T0 (Log CFU/mL)	G3h (Log CFU/mL)	Gastric Survival (%)	I4h (Log CFU/mL)	Intestinal Transition (%)	Cumulative GIT Survival (%)	Log Reduction
2BP2Ce2	7.15	6.70	35.48	6.44	54.95	19.50	0.71
3BP3Du1	7.78	7.39	40.74	6.36	9.33	3.80	1.42
2BP1Ce1	6.91	6.64	53.70	6.52	75.86	40.74	0.39
1BP2Fe	6.79	5.75	9.12	5.50	56.23	5.13	1.29
1BP2Ce1	6.80	6.65	70.79	6.48	67.61	47.86	0.32
1BP3Je	5.99	5.68	48.98	5.50	66.07	32.36	0.49
1BP1Co1	7.18	7.05	74.13	6.60	35.48	26.30	0.58

Note: Under a ≤1.0 log_10_ reduction criterion (FAO/WHO-aligned and the field standard for sequential GI tolerance assays), five isolates qualify: 2BP1Ce1, 1BP2Ce1, 1BP3Je, 1BP1Co1, and 2BP2Ce2.

**Table 2:** The results of OD_570_ measurements from the adhesion assay of *Bacillus* spp. isolates.

Sample	Isolate	*n*	OD_570_ Range	Mean OD_570_ ± SD	OD_570_ Corrected	Relative Adhesion (%)
Cecum (Ce)	Ce01 p1	9	0.212–0.218	0.215 ± 0.003	0.164	9.909
	Ce02 p1	9	0.208–0.214	0.211 ± 0.003	0.160	9.668
	Ce03 p1	9	0.227–0.235	0.231 ± 0.004	0.180	10.876
	Ce04 p1	9	0.185–0.209	0.197 ± 0.012	0.146	8.822
	Ce01 p2	9	0.190–0.200	0.195 ± 0.005	0.144	8.701
	Ce02 p2	9	0.103–0.121	0.112 ± 0.009	0.061	3.686
	Ce06 p2	9	0.203–0.219	0.211 ± 0.008	0.160	9.668
	1BP2Ce1	9	1.569–1.575	1.572 ± 0.002	1.521	91.903
	2BP1Ce1	9	1.382–1.386	1.384 ± 0.002	1.333	80.544
	2BP2Ce2	9	1.211–1.213	1.212 ± 0.001	1.161	70.151
Mid-colon (Co)	Co04 p2	9	0.224–0.238	0.231 ± 0.007	0.180	10.876
	Co05 p2	9	0.151–0.157	0.154 ± 0.003	0.103	6.224
	Co06 p2	9	0.214–0.236	0.225 ± 0.011	0.174	10.514
	Co07 p2	9	0.224–0.248	0.236 ± 0.012	0.185	11.178
	1BP1Co1	9	1.703–1.708	1.706 ± 0.002	1.655	100
Ileum (IL)	il02 p1	9	0.164–0.186	0.175 ± 0.011	0.124	7.492
	il03 p1	9	0.207–0.221	0.214 ± 0.007	0.163	9.849
	il04 p1	9	0.229–0.239	0.234 ± 0.005	0.183	11.057
	il01 p2	9	0.204–0.220	0.212 ± 0.008	0.161	9.728
	il02 p2	9	0.207–0.219	0.213 ± 0.006	0.162	9.789
	il03 p2	9	0.113–0.117	0.115 ± 0.002	0.064	3.867
	il04 p2	9	0.162–0.170	0.166 ± 0.004	0.115	6.949
	il05 p2	9	0.204–0.226	0.215 ± 0.011	0.164	9.909
	il06 p2	9	0.201–0.229	0.215 ± 0.014	0.164	9.909
	il07 p2	9	0.115–0.133	0.124 ± 0.009	0.073	4.412
	il08 p2	9	0.198–0.208	0.203 ± 0.005	0.152	9.184
	3BP2IL	9	1.476–1.488	1.482 ± 0.006	1.431	86.465
Feces (Fe)	Fe02 p1	9	0.215–0.227	0.221 ± 0.006	0.170	10.272
	Fe01 p2	9	0.089–0.105	0.097 ± 0.008	0.046	2.779
	Fe02 p2	9	0.209–0.233	0.216 ± 0.007	0.165	9.970
	Fe05 p2	9	0.155–0.179	0.167 ± 0.012	0.116	7.009
	Fe06 p2	9	0.205–0.227	0.216 ± 0.011	0.165	9.970
	Fe09 p2	9	0.204–0.218	0.211 ± 0.007	0.160	9.668
	Fe11 p2	9	0.240–0.268	0.254 ± 0.014	0.203	12.266
	1BP2Fe	9	1.371–1.375	1.373 ± 0.002	1.322	79.879
	2BP4Fe	9	1.287–1.299	1.293 ± 0.006	1.242	75.045
Duodenum (Du)	Du01 p2	9	0.146–0.158	0.152 ± 0.006	0.101	6.103
	Du02 p2	9	0.102–0.126	0.114 ± 0.012	0.063	3.807
	3BP3Du1	9	1.535–1.541	1.538 ± 0.003	1.487	89.849
Jejunum (Je)	Je03 p1	9	0.178–0.202	0.190 ± 0.012	0.139	8.399
	Je01 p2	9	0.221–0.223	0.222 ± 0.001	0.171	10.332
	Je02 p2	9	0.136–0.148	0.142 ± 0.006	0.091	5.498
	Je03 p2	9	0.125–0.151	0.138 ± 0.013	0.087	5.257
	Je04 p2	9	0.239–0.243	0.241 ± 0.002	0.190	11.480
	Je05 p2	9	0.107–0.129	0.118 ± 0.011	0.067	4.048
	Je06 p2	9	0.248–0.258	0.253 ± 0.005	0.202	12.205
	Je07 p2	9	0.237–0.245	0.241 ± 0.004	0.190	11.480
	Je08 p2	9	0.204–0.226	0.215 ± 0.011	0.164	9.909
	1BP3Je	9	1.379–1.391	1.385 ± 0.006	1.334	80.604
	3BP3Je	9	1.480–1.484	1.482 ± 0.002	1.431	86.465

**Table 3:** Genome assembly and annotation statistics of the 1BP1Co1 isolate.

Genome Characteristic	Value
Total genome size (bp)	4,148,036
Genome coverage	242X
Genome topology	Circular
GC content (%)	43.65
Number of contigs	1
N50 (bp)	4,148,036
Largest contig size (bps)	4,148,036
Genome completeness (%)	99.10%
Contamination (%)	0.16
Protein coding sequences (CDSs)	4275
rRNAs	30
tRNAs	86

**Table 4:** Identified secondary metabolite biosynthetic gene clusters (BGCs) from antiSMASH v8.0.4.

Region	BGC Type	From (bp)	To (bp)	Similarity Confidence	MIBiG Reference Accession	Most Similar Known Cluster	
1	Terpene	204,123	224,926	—	—	—	—
2	transAT-PKS, PKS-like, T3PKS, NRPS	817,296	932,071	High	BGC0001089	Bacillaene	NRPS: Type I + PKS: Type I
3	NRPS, betalactone	1,002,763	1,080,524	High	BGC0001095	Fengycin	NRPS: Type I
4	Terpene	1,143,965	1,165,863	—	—	—	—
5	T3PKS	1,343,984	1,385,081	Low	BGC0001847	1-Carbapen-2-em-3-carboxylic acid	Other: other
6	Terpene-precursor	1,562,853	1,583,743	—	—	—	—
7	NRP-metallophore, NRPS	2,266,208	2,317,985	High	BGC0000309	Bacillibactin	NRPS: Type I
8	Sactipeptide	2,843,178	2,864,789	High	BGC0000602	Subtilosin A	Ribosomal: RiPP: Thiopeptide
9	Other	2,871,818	2,913,236	High	BGC0001184	Bacilysin	Other: other
10	NRPS	3,582,436	3,647,827	High	BGC0000433	Surfactin	NRPS: Type I
11	Lanthipeptide-class-III	3,747,041	3,773,370	—	—	—	—

— Indicates no significant KnownClusterBlast hit. Abbreviations: NRPS, non-ribosomal peptide synthetase; PKS, polyketide synthase; transAT-PKS, trans-acyltransferase polyketide synthase; T3PKS, Type III polyketide synthase; RiPP, ribosomally synthesized and post-translationally modified peptide.

## Data Availability

The genome assembly generated and analyzed in this study has been deposited in the National Center for Biotechnology Information (NCBI) database under assembly accession GCF_057443935.1 (RefSeq; GenBank accession GCA_057443935.1), assembly name ASM5744393v1, and is publicly accessible at https://www.ncbi.nlm.nih.gov/datasets/genome/GCF_057443935.1/, accessed on 12 January 2025. The *Bacillus* strains characterized in this study are available from the corresponding author upon reasonable request. All other datasets supporting the conclusions of this article are included within the article and its additional file.

## Supplementary Materials

The following supporting information can be downloaded at https://www.mdpi.com/article/10.3390/ani16162449/s1. Table S1. The total number of isolates obtained from the intestinal and fecal samples of pigs. Table S2. The results of the hemolytic activity test of Bacillus spp. isolates obtained from cultivation on tryptone soya agar (TSA). Table S3. OD_600_ values of pathogenic *Escherichia coli* groups following treatment with cell-free culture supernatants (CFCSs) from selected *Bacillus* spp. isolates. Table S4. Molecular identification of selected *Bacillus* isolates based on 16S rRNA gene sequencing. Species-level identification was achieved by amplifying and sequencing the 16S rRNA gene. The resulting sequences were compared against established reference type strains to determine percentage similarity. Table S5. Digital DNA–DNA hybridization (dDDH) analysis of *Bacillus* spp. Table S6. In silico prediction of antimicrobial resistance and virulence-associated genes in the *Bacillus subtilis* 1BP1Co1 genome. Figure S1. Effect of *Bacillus subtilis* 1BP1Co1 on IPEC-J2 cell viability.
