# Supplementary material for: Phenotypic and Genomic Analysis of Swine-Derived Bacillus subtilis 1BP1Co1 as a Functional Probiotic Candidate to Mitigate Escherichia coli-Associated Diarrhea
Source: Animals (Basel). 2026 Aug 7;16(16):2449. doi: 10.3390/ani16162449 (PMC13508853; doi:10.3390/ani16162449)
Supplement: Supplementary file 1 [file animals-16-02449-s001.zip › animals-4347910-supplementary.pdf]

Table S1. The total number of isolates obtained from the intestinal and fecal samples of pigs.

**Sample**	***Bacillus* isolate**
Duodenum	4
Jejunum	2
Ileum	10
Cecum	24
Mid – colon	9
Feces	10
**Total**	59

Table S2. The results of the hemolytic activity test of *Bacillus* spp. isolates obtained from cultivation on tryptone soya agar (TSA).

Samples	Isolates	Hemolytic activity
Duodenum (Du)	BDu01 P0	Gamma hemolysis
	BDu01 P1	
	2BP4Du	
	3BP3Du	
Jejunum (Je)	1BP3Je	
	3BP3Je	
Ileum (IL)	BIL01 P1	
	1BP1IL	
	1BP2IL	
	1BP3IL	
	2BP1IL	Beta hemolysis
	2BP2IL	
	2BP3IL	
	3BP1IL	Gamma hemolysis
	3BP2IL	Alpha hemolysis
	3BP3IL	Gamma hemolysis
Cecum (Ce)	BCe01 P0	
	BCe02 P0	
	BCe03 P0	
	BCe04 P0	
	BCe05 P0	
	BCe0 P1	
	BCe01 P1	
	1BP1Ce	
	1BP2Ce1	
	1BP2Ce2	
	1BP3Ce1	
	1BP3Ce2	
	2BP1Ce1	Beta hemolysis
	2BP2Ce1	Alpha hemolysis
	2BP2Ce2	Gamma hemolysis
	2BP3Ce1	
	2BP3Ce2	
	2BP4Ce1	
	2BP4Ce2	
	3BP1Ce	Alpha hemolysis
	3BP2Ce1	Beta hemolysis
	3BP2Ce2	
	3BP3Ce1	Alpha hemolysis
	3BP3Ce2	
Mid-colon (Co)	BCo01 P0	
	BCo01 P1	
	1BP1Co	Gamma hemolysis
	2BP1Co	Beta hemolysis
	2BP2Co	
	2BP3Co	

Samples	Isolates	Hemolytic activity
	3BP1Co	
	3BP2Co	
	3BP3Co	
Feces (Fe)	BFe01 P0	Gamma hemolysis
	BFe01 P1	
	BFe02 P1	
	1BP2Fe	Gamma hemolysis
	1BP3Fe	
	2BP1Fe	Beta hemolysis
	2BP2Fe	
	2BP4Fe	Gamma hemolysis
	3BP2Fe	Alpha hemolysis
	3BP3Fe	Gamma hemolysis

Table S3. OD_{600} values of pathogenic *Escherichia coli* groups following treatment with cell-free culture supernatants (CFCSs) from selected *Bacillus* spp. isolates.

1BP2Ce1

CFCS (%)	EAEC	EHEC	EIEC	ETEC	EPEC
100	0.073	0.057	0.054	0.073	0.058
50	0.098	0.094	0.072	0.253	0.083
25	0.189	0.119	0.238	0.295	0.184
12.5	0.225	0.179	0.329	0.351	0.259
6.25	0.264	0.185	0.384	0.399	0.341
Growth Control	0.287	0.196	0.428	0.448	0.369

2BP1Ce1

CFCS (%)	EAEC	EHEC	EIEC	ETEC	EPEC
100	0.058	0.053	0.032	0.052	0.043
50	0.073	0.079	0.043	0.073	0.072
25	0.093	0.154	0.063	0.092	0.174
12.5	0.179	0.178	0.245	0.285	0.275
6.25	0.261	0.194	0.364	0.375	0.362
Growth Control	0.287	0.196	0.428	0.448	0.369

2BP2Ce2

CFCS (%)	EAEC	EHEC	EIEC	ETEC	EPEC
100	0.168	0.145	0.036	0.035	0.042
50	0.194	0.156	0.079	0.067	0.064
25	0.238	0.164	0.274	0.082	0.081
12.5	0.259	0.174	0.385	0.284	0.274
6.25	0.288	0.189	0.425	0.355	0.312
Growth Control	0.287	0.196	0.428	0.448	0.369

1BP1Co

CFCS (%)	EAEC	EHEC	EIEC	ETEC	EPEC
100	0.035	0.036	0.042	0.063	0.035
50	0.061	0.051	0.062	0.098	0.063
25	0.083	0.072	0.094	0.286	0.094
12.5	0.184	0.093	0.245	0.385	0.276
6.25	0.229	0.184	0.385	0.440	0.319
Growth Control	0.287	0.196	0.428	0.448	0.369

3BP2IL

CFCS (%)	EAEC	EHEC	EIEC	ETEC	EPEC
100	0.137	0.125	0.183	0.037	0.036
50	0.159	0.138	0.263	0.092	0.053
25	0.184	0.164	0.302	0.193	0.072
12.5	0.235	0.174	0.384	0.248	0.094
6.25	0.275	0.186	0.428	0.374	0.287
Growth Control	0.287	0.196	0.428	0.448	0.369

1BP2Fe

CFCS (%)	EAEC	EHEC	EIEC	ETEC	EPEC
100	0.139	0.047	0.257	0.047	0.015
50	0.183	0.082	0.286	0.071	0.043
25	0.201	0.148	0.341	0.091	0.061
12.5	0.239	0.174	0.374	0.284	0.089
6.25	0.267	0.183	0.411	0.354	0.294
Growth Control	0.287	0.196	0.428	0.448	0.369

2BP4Fe

CFCS (%)	EAEC	EHEC	EIEC	ETEC	EPEC
100	0.072	0.042	0.042	0.062	0.058
50	0.091	0.061	0.082	0.091	0.093
25	0.174	0.092	0.184	0.286	0.184
12.5	0.199	0.165	0.269	0.380	0.253
6.25	0.263	0.194	0.377	0.422	0.337
Growth Control	0.287	0.196	0.428	0.448	0.369

3BP3Du1

CFCS (%)	EAEC	EHEC	EIEC	ETEC	EPEC
100	0.148	0.134	0.268	0.061	0.044
50	0.182	0.159	0.321	0.092	0.067
25	0.249	0.168	0.371	0.295	0.184
12.5	0.261	0.179	0.408	0.339	0.295
6.25	0.281	0.183	0.426	0.401	0.342
Growth Control	0.287	0.196	0.428	0.448	0.369

1BP3Je

CFCS (%)	EAEC	EHEC	EIEC	ETEC	EPEC
100	0.023	0.128	0.173	0.024	0.189
50	0.047	0.147	0.206	0.052	0.248
25	0.071	0.162	0.268	0.074	0.291
12.5	0.094	0.173	0.349	0.088	0.321
6.25	0.195	0.189	0.401	0.397	0.358
Growth Control	0.287	0.196	0.428	0.448	0.369

3BP3Je

CFCS (%)	EAEC	EHEC	EIEC	ETEC	EPEC
100	0.148	0.138	0.248	0.034	0.053
50	0.173	0.159	0.293	0.046	0.089
25	0.201	0.166	0.329	0.071	0.194
12.5	0.239	0.174	0.372	0.083	0.257
6.25	0.262	0.185	0.405	0.294	0.302
Growth Control	0.287	0.196	0.428	0.448	0.369

Note: MIC was defined as the lowest nominal CFCS dilution level showing no visible bacterial growth after 18–24 h of incubation. OD_{600} values were used as supporting data for MIC interpretation together with visual growth assessment and control wells. Growth control represents pathogenic *Escherichia coli* cultured without CFCS. “>100” indicates that complete inhibition was not observed at the highest CFCS level tested.

Table S4. Molecular identification of selected Bacillus isolates based on 16S rRNA gene sequencing. Species-level identification was achieved by amplifying and sequencing the 16S rRNA gene. The resulting sequences were compared against established reference type strains to determine percentage similarity.

Samples	Isolates	Species	Type strain	Similarity (%)
Duodenum (Du)	3BP3Du1	*Bacillus siamensis*	KCTC 13613	99.71
Jejunum (Je)	1BP3Je	*Bacillus velezensis*	CR-502	92.25
	3BP3Je	*Bacillus siamensis*	KCTC 13613	92.82
Ileum (IL)	3BP2IL	*Bacillus velezensis*	CR-502	100.00
Cecum (Ce)	1BP2Ce1	*Bacillus siamensis*	KCTC 13613	99.50
	2BP1Ce1	*Bacillus siamensis*	KCTC 13613	99.14
	2BP2Ce2	*Bacillus siamensis*	KCTC 13613	99.86
Mid-colon (Co)	1BP1Co1	*Bacillus siamensis*	KCTC 13613	99.93
Feces (Fe)	1BP2Fe	*Bacillus velezensis*	CR-502	92.26
	2BP4Fe	*Bacillus siamensis*	KCTC 13613	99.86

Table S5. Digital DNA–DNA hybridization (dDDH) analysis of *Bacillus spp.*

Query strain	Subject strain	dDDH (d0, in %)	C.I. (d0, in %)	dDDH (d4, in %)	C.I. (d4, in %)	dDDH (d6, in %)	C.I. (d6, in %)	G+C content difference (in %)
1BP1Co1	*Bacillus subtilis* ATCC 6051	93.8	[91.2 - 95.6]	86.7	[84.1 - 88.9]	94.9	[93.0 - 96.3]	0.13
1BP1Co1	*Bacillus subtilis* NCIB 3610	92.4	[89.5 - 94.5]	86.5	[83.8 - 88.7]	93.8	[91.7 - 95.5]	0.26
1BP1Co1	*Micromonospora provocatoris* MT25	13.1	[10.4 - 16.4]	84.8	[82.1 - 87.2]	13.6	[11.2 - 16.4]	28.19
1BP1Co1	*Bacillus stercoris* D7XPN1	90.5	[87.3 - 92.9]	62.7	[59.8 - 65.5]	88.1	[85.1 - 90.5]	0.15
1BP1Co1	*Bacillus spizizenii* TU-B-10	86.8	[83.2 - 89.7]	51.1	[48.4 - 53.7]	81.6	[78.2 - 84.5]	0.18
1BP1Co1	*Bacillus inaquosorum* KCTC 13429	84.1	[80.3 - 87.2]	50.3	[47.6 - 52.9]	79	[75.6 - 82.1]	0.04
1BP1Co1	*Bacillus rugosus* SPB7	82.4	[78.5 - 85.7]	49.2	[46.5 - 51.8]	77.2	[73.8 - 80.4]	0.53
1BP1Co1	*Bacillus cabrialesii* TE3	87.6	[84.1 - 90.4]	47.9	[45.3 - 50.5]	81.1	[77.7 - 84.1]	0.47
1BP1Co1	*Bacillus cabrialesii* subsp. *tritici* TSO2	85.3	[81.6 - 88.4]	47.9	[45.4 - 50.5]	79.2	[75.8 - 82.2]	0.36
1BP1Co1	*Bacillus tequilensis* NCTC13306	66	[62.1 - 69.6]	45.5	[42.9 - 48.1]	62.5	[59.2 - 65.7]	0.33
1BP1Co1	*Bacillus vallismortis* DV1-F-3	67.2	[63.3 - 70.8]	42.9	[40.4 - 45.5]	62.6	[59.3 - 65.8]	0.11
1BP1Co1	*Bacillus mexicanus* FSQ1	67.8	[63.9 - 71.4]	40	[37.5 - 42.6]	61.9	[58.6 - 65.1]	0.69

Table S6. *In silico* prediction of antimicrobial resistance and virulence-associated genes in the *Bacillus subtilis* 1BP1Co1 genome.

Database	*Bacillus_subtilis*_mprF	aadK	blt	bmr	lmrB	mphK	rphB	rphC	tmrB	vmlR	ykkC	ykkD
card	100	100	100	100	100	100	97.78	.	100	100	100	100
ncbi	.	100	.	.	.	100	.	99.35	.	100	.	.
resfinder	.	100	.	.	.	100	.	.	.	.	.	.
vfdb	.	.	.	.	.	.	.	.	.	.	.	.

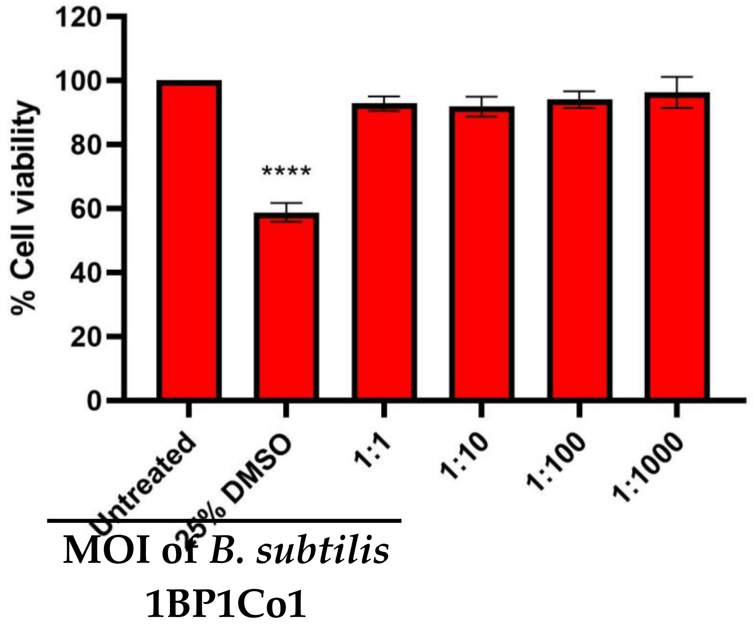

Figure S1. Effect of *Bacillus subtilis* 1BP1Co1 on IPEC-J2 cell viability. Percentage of cell viability of the IPEC-J2 porcine enterocyte cell line following exposure to different multiplicities of infection (MOI) of B. subtilis 1BP1Co1 for 6 hours. Treatment with 25% DMSO served as a positive control for cytotoxicity. Data are represented as means ± SEM. ***p* < 0.01 indicates a statistically significant difference compared to the untreated control.
